# Testing the Multi-Theory Model (MTM) to Predict the Use of New Technology for Social Connectedness in the COVID-19 Pandemic

**DOI:** 10.3390/healthcare9070838

**Published:** 2021-07-01

**Authors:** Manoj Sharma, Kavita Batra, Jason Flatt

**Affiliations:** 1 Department of Environmental and Occupational Health, University of Nevada, Las Vegas, NV 89119, USA; Manoj.Sharma@unlv.edu (M.S.); Jason.flatt@unlv.edu (J.F.); 2 Office of Research, Kirk Kerkorian School of Medicine, University of Nevada, Las Vegas, NV 89102, USA

**Keywords:** social isolation, social connectedness, loneliness, depression, technology, internet, smartphones, m-health, COVID-19, pandemic

## Abstract

Loneliness or social isolation, recently described as a “behavioral epidemic,” remains a long-standing public health issue, which has worsened during the COVID-19 pandemic. The use of technology has been suggested to enhance social connectedness and to decrease the negative health outcomes associated with social isolation. However, till today, no theory-based studies were performed to examine the determinants of technology use. Therefore, the current study aims to test theory-based determinants in explaining the adoption of new technology in a nationally representative sample during the COVID-19 pandemic (*n* = 382). A psychometrically reliable and valid instrument based on the multi-theory model (MTM) of health behavior change was administered electronically using a cross-sectional study design. A total of 47.1% of the respondents reported high levels of social isolation, and 40.6% did not use any new technology. Among technology users (59.4%), the three initiation constructs participatory dialogue (b = 0.054, *p* < 0.05), behavioral confidence (b = 0.184, *p* < 0.001), and changes in the physical environment (b= 0.053, *p* < 0.05) were significant and accounted for 38.3% of the variance in the initiation of new technologies. Concerning sustenance in technology users, all three constructs emotional transformation (b = 0.115, *p* < 0.001), practice for change (b = 0.086, *p* < 0.001), and changes in the social environment (b = 0.061, *p* < 0.001) were significant and accounted for 42.6% of the variance in maintaining the use of new technology. MTM offers a powerful framework to design health promotion interventions encouraging the use of new technologies to foster greater social connectedness amid the COVID-19 pandemic and beyond it.

## 1. Introduction

Loneliness or perceived social isolation were recently described as a “behavioral epidemic,” which has worsened in the wake of the COVID-19 pandemic [1,2,3]. Loneliness reflects subjective experiences, while social isolation describes the objective state of an individual’s social interactions [4,5]. Research has shown that loneliness and social isolation have adverse physical and mental health outcomes. Loneliness and social isolation are associated with an increased risk of depression, cognitive decline, heart disease, stroke, and premature mortality [6,7,8]. A meta-analysis found that both subjective and objective loneliness or social isolation increases the risk of mortality, with a 26% increased likelihood of mortality for individuals reporting loneliness, 29% for those reporting social isolation, and 32% for those living alone [7]. Moreover, the risk of mortality following loneliness/social isolation was equivalent to the mortality risk among individuals with extreme or severe obesity.

The COVID-19 pandemic has heightened both the public’s and public health practitioners’ concerns about loneliness and social isolation. Specifically, stay-at-home orders and social distancing measures to reduce the spread of COVID-19 have resulted in people avoiding public spaces and crowds, canceling social activities, and avoiding close contact with others. These preventive behaviors are essential for those at a greater risk of severe illness from COVID-19 and related hospitalization and mortality [9]. Individuals at higher risk for severe illness, those with pre-existing conditions (hypertension, pulmonary disease, diabetes, and cardiovascular disease), racial/ethnic minorities, older age, and male sex, may also be more likely to experience loneliness and social isolation [10]. Studies have suggested that COVID-19 preventive behaviors may result in greater odds of reporting loneliness and social isolation [11,12,13]. For instance, a population-based study in the United States (U.S.) examining the impact of COVID-19 social distancing and preventive behaviors found that 54% of participants reported loneliness [14]. Loneliness was associated with more significant depressive symptoms among people with fewer social interactions than those who had more frequent in-person social interactions or connections [14].

Given the COVID-19 pandemic and the need for continued social distancing and preventative measures, novel ways to promote social connectedness and reduce feelings of loneliness are greatly needed. New technologies have been proposed as one way to counter social distancing and stay-at-home orders while encouraging social interactions and social connectedness [15]. Studies examining COVID-19 preventive behaviors and technology use suggest that novel technologies may promote social connectedness and reduce feelings of loneliness [16,17]. However, there is a need for a theory-driven approach to aid understanding of factors associated with new technology and ways that promote the technology use to improve social connectedness during the COVID-19 pandemic.

The Multi-Theory Model (MTM) of health behavior change is a unique theory that can be utilized to explain the factors related to both initiating and sustaining new health behaviors [18]. Three constructs of MTM represent the initiation phase of behavior change, including participatory dialogue (advantages offsetting the disadvantages of the health behavior change), behavioral confidence (beliefs that one can perform the behavior change), and changes in the physical environment (having resources at one’s disposal for the behavior change). Sustenance includes the following constructs: emotional transformation (translating feelings into goals for the behavior change), practice for change (creating new habits that support the health behavior change), and changes in the social environment (obtaining social support to help one maintain the health behavior change). Previous studies have shown that the MTM of health behavior change is effective in promoting and sustaining a variety of health behaviors, including handwashing, physical activity, portion sizes, consuming water instead of sugar-sweetened beverages, and potentially increasing the uptake of technology [19,20,21,22,23].

To our knowledge, no studies have investigated the use of MTM or related theories in promoting technology use among populations at risk for loneliness and social isolation due to the COVID-19 pandemic. This study explores the determinants of new technology use for promoting social connectedness during the COVID-19 pandemic by utilizing the conceptual paradigm of MTM. Specifically, we investigated whether the factors related to both the initiation and sustainability constructs of MTM would be associated with new technology use during the COVID-19 pandemic in a nationally representative sample of adults in the United States.

## 2. Materials and Methods

### 2.1. Study Design and Data Collection

This cross-sectional study collected data from 22 February 2021 to 25 February 2021 through Qualtrics utilizing a high-quality panel of participants. Available online: https://www.qualtrics.com/research-services/online-sample/). The general information to use Qualtrics panel platforms has been described by Miller and colleagues [24].

### 2.2. Eligibility Criteria:

The sample was recruited through Qualtrics to include U.S. residents aged 18 years or above with a sufficient understanding of the English language. A priori quota sampling was established to recruit a targeted sample. Quota sampling was performed to recruit a sample that mirrored Census representation by sex, race, and ethnicity. Sampling quotas for age and regional/geographical distribution were not used for sampling.

### 2.3. Ethical Considerations

The study (protocol # 1721549-1) was considered an exempt research study by the Institutional Review Board (IRB). Participation in the study was voluntary, and details about the study’s objectives and significance were provided to participants before completing the survey. Personal identifiers were not collected to ensure anonymity. Multiple responses from the same participants were restricted by enforcing the Ballot Box Stuffing option. In other words, only one response per participant was allowed. Quality checks were performed to exclude responses completed in less than 2 min (reflective of participants not responding thoughtfully).

### 2.4. Data Protection and Information Security

This study utilized data obtained through a contractual agreement between the principal investigator (PI) and Qualtrics Research Services group. As an essential part of the contract, all data privacy laws and regulations were followed by both parties. Qualtrics research services do not allow the collection of any respondent’s personal information. All personal identifiers were completely removed to maintain confidentiality. All electronic files of de-identified data were kept secure within the institution file storage network and regularly backed up to an encrypted and password-protected external hard drive, stored in a locked safe in a locked office of the researchers. Only researchers approved by this proposed protocol had access to the file storage network that housed these data. Desktop computers and user logins associated with this study were password-protected.

### 2.5. Survey Questionnaire

**Figure 1:**
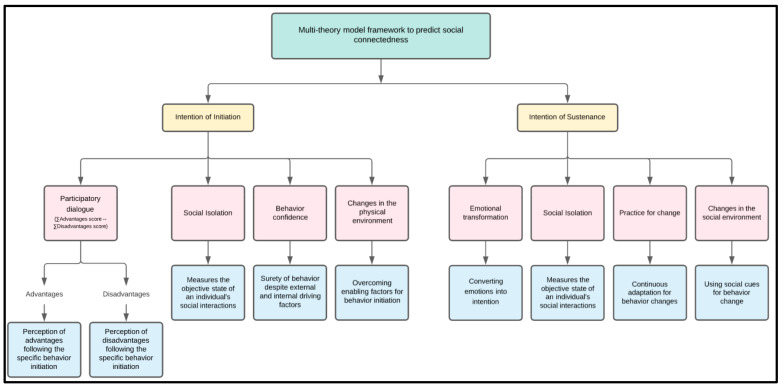
Flowchart detailing multi-theory model framework to predict social connectedness.

As guided by MTM, a 40-item survey questionnaire was developed to measure the use and acceptance of new technologies for improving social connectedness during the COVID-19 pandemic. The survey comprised 14 items related to demographic background, 3 items for social isolation, and 23 items for the two primary MTM theoretical constructs (initiation and sustenance). The face and content validity of the questionnaire was assessed by a panel of 6 subject matter experts (SMEs), who provided feedback to improve the survey. The panel review was blinded, meaning SMEs were not aware of other’s input on the survey. A total of 23 changes/clarifications, primarily to improve readability, were incorporated in the instrument between rounds 1 and 2 of the SMEs’ review. The questionnaire was reviewed 3 times after incorporating SMEs’ feedback before dissemination of the survey. Detailed information about MTM constructs (initiation, sustenance, and social isolation) is shown in Figure 1. All constructs of initiation and sustenance were measured on a 5-point Likert scale [18]. To examine social isolation during the COVID-19 pandemic, 3 items were used to assess. The summative score of 3 social isolation items ranged from 1–12 units, and a higher score indicated more social isolation. The instrument was developed using clear and appropriate language corresponding to the Flesch reading ease of 66.0 and Flesch-Kincaid Grade Level of 6.7 grade [18,25].

### 2.6. Statistical Analysis

Participants’ responses to Qualtrics were exported to a spreadsheet and then imported to IBM SPSS version 27.0 (IBM Corp. Armonk, NY, USA) for analysis. Confirmatory factor analysis (CFA) using the extraction method of maximum likelihood was utilized. Reliability diagnostics or Cronbach’s alpha was computed for all the subscales. Critical values for determining one-factor solution were set according to the prespecified literature’s criteria [26]. The critical value for a correlation coefficient at α = 0.01 for a 2-tailed test for the sample size of 400 participants was 0.129. This was doubled for testing the significance of loading [26]. Hence, a critical value of 0.258 was deemed appropriate [26]. The normality assumption of data was assessed using the Shapiro–Wilk test and normal Q-Q plots. An independent-samples- *t*-test was utilized to compare the mean scores across new technology users and non-user groups. A chi-square test was conducted to compare categorical variables. A post-hoc contingency table analysis using adjusted residuals (or Z scores) was performed in case of multiple comparisons. Bonferroni corrected *p*-values were generated. Bootstrapped significance testing for the chi-square test was conducted to examine replicability and consistency. The score of social isolation was dichotomized as low social isolation (≤6.0) and high social isolation (>6.0) by using the median-split method [27]. Categorical variables were expressed as counts and proportions, whereas continuous variables were represented as means and standard deviations. Two separate Hierarchical Regression Models (HRM) were built to predict the variance in the likelihood of initiation and sustenance of new technology behavior by multiple factors, such as demographic characteristics, social isolation, and MTM constructs. All assumptions of HRM were assessed. The significance level was set at 0.05, and 95% confidence intervals were reported wherever applicable.

### 2.7. Testing of HRM Assumptions

Our data meet all the 8 assumptions of HRM, which were as follows:

**Assumption** **#** **1:** *The dependent variables of this study (initiation and sustenance) were measured on a continuous scale.*

**Assumption** **#** **2:** *There were 2 or more independent variables, which were measured either at continuous (initiation and sustenance constructs) or nominal level (demographic variables).*

**Assumption** **#** **3:** *There was a linear relationship between the continuous independent and dependent variables as assessed by partial regression plots.*

**Assumption** **#** **4:** *There was independence of residual errors as assessed by a Durbin–Watson statistic.*

**Assumption** **#** **5:** *No multicollinearity between the variables was assessed.*

**Assumption** **#** **6:** *There were no significant outliers, as no data point was above 3 standard deviations.*

**Assumption** **#** **7:** *The errors (residuals) were normally distributed, as assessed by a Q-Q plot.*

**Assumption** **#** **8:** *There was homoscedasticity of residuals as assessed by visual inspection of a plot between residual versus predicted values.*

### 2.8. Sample Size Justification

Priori power analysis was conducted to determine sample size using G* Power statistical software. The sample sizes for independent-samples t-test and chi-square analysis were estimated depending upon Cohen’s effect sizes conventions [28,29]. The total sample size estimated with a power of 0.99 was *n* = 254 for the *t*-test, *n* = 297 for the Chi-square test, and *n* = 146 for the regression analysis using the effect sizes of 0.5, 0.3, and 0.15, respectively. The sample size with the greatest value (*n* = 297) was considered appropriate given it satisfied the minimum requirement of all statistical tests proposed. After factoring in 25% oversampling to offset missing values, our minimum sample requirement was *n* = 371.

## 3. Results

### Sample Characteristics

The survey was completed by a total of 382 participants. Only five responses (1.8%) were incomplete and were deleted (case-wise) from the study. Among the 382 participants, the distribution was comparable among sex categories (50.3% females vs. 49.5% males, Table 1). The mean age of the sample was 43.9 ± 18.3 years. The sample was predominantly White (71.2%, *n* = 272) and non-Hispanic (82.7%, *n* =316; Table 1). Nearly 25% (99 of 382) of participants had a yearly income of less than $25,000. Nearly a third of participants reported being “never married” (Table 1). Of 382 participants, 202 (52.9%) used new technology during the COVID-19 pandemic, and video conferencing was the most commonly used technology in combination with other technologies. More than 50% of the sample population had a higher social isolation score indicative of loneliness (Table 1). Participants who reported new technology use were younger (<55 years of age) (73.1% vs. 26.9%; *p* = 0.02), non-Hispanic/Latino (78.9% vs. 21.1%; *p* = 0.02), employed (56.4% vs. 43.6%; *p* < 0.0001), had an income over $125,000 (12.3% vs. 3.2%; *p* < 0.0001), had health insurance (88.5% vs. 11.5%; *p* < 0.0001), were socially isolated (54.6% vs. 45.4%; *p* < 0.0001), and more likely to access smartphones with internet (Table 2).

Except for the score of disadvantages, there were significant differences in the mean scores for all constructs of initiation and sustenance among technology users and non-users (Table 3). Technology users had a statistically significant higher mean scores for initiation compared to technology non-users (2.72 ± 1.2 vs. 1.82 ± 1.3, 95% Confidence Interval [−1.151, −0.646], *p* < 0.0001, Table 3). Similarly, the mean score for sustenance was higher among technology users compared to non-users (2.78 ± 1.09 vs. 1.99 ± 1.23, 95% CI [−1.028, −0.544], *p* < 0.0001, Table 3). Participants who used new technology were more likely to report social isolation than technology non-users (M = 6.96 vs. 5.51; *p* < 0.0001 with a mean difference of 1.46 [95% CI: 0.784, 2.13].

Two separate hierarchical multiple regression models were utilized to predict the variance in initiation and sustenance of the behavior by MTM constructs beyond demographic variables among technology users and non-users (Table 4). Among participants using technology during a pandemic, the full model (Model 4) to predict initiation was statistically significant, R^2^ = 0.408, F (9216) = 16.545, *p* < 0.0001; adjusted R^2^ = 0.383 (Table 4). All MTM constructs added statistical significance to the prediction. The standardized regression coefficient value indicated that the behavior confidence was associated with the maximum increase of 0.455 points on the initiation score (Table 4). Similarly, for sustenance model, the Model 4 was statistically significant and improved prediction, *R*^2^ = 0.449, *F* (9216) = 19.546, *p* < 0.0001; adjusted *R*^2^ = 0.426 (Table 4). The value of the standardized regression coefficient in the sustenance model indicated that the emotional transformation was associated with the maximum increase of 0.326 points on the initiation score among technology users (Table 4).

Among participants not using new technology during the pandemic (Model 4), initiation was statistically significant, *R*^2^ = 0.430, *F* (9, 145) = 12.178, *p* < 0.0001; adjusted *R*^2^ = 0.395 (Table 5). In addition, in a regression analysis with sustenance as a dependent variable, the full model (Model 4) was statistically significant, *R*^2^ = 0.513, *F* (9, 145) = 16.941, *p* < 0.0001; adjusted *R*^2^ = 0.482 (Table 5). The value of standardized regression coefficients indicated that the changes in the physical environment were associated with an increase of 0.300 units on the initiation score among technology non-users (Table 5). Regarding sustenance, changes in the social environment were associated with an increase of 0.393 units in the sustenance among technology non-users (Table 5).

A confirmatory factor analysis (CFA) was performed on eight theoretical constructs (7 MTM construct and 1 social isolation) to establish construct validity of the subscales. The suitability of CFA was assessed before the analysis. Bartlett’s Test of Sphericity was statistically significant (*p* < 0.0005), indicating that the data were likely factorizable. Inspection of the correlation matrix indicated that all variables had at least one correlation coefficient greater than 0.3. The overall Kaiser–Meyer–Olkin (KMO) measure was 0.87, which classifies as “middling” to “meritorious”, according to Kaiser [30]. CFA revealed that all MTM constructs (advantages, disadvantages, behavior confidence, changes in the physical environment, emotional transformation, practice for change, changes in the social environment, and construct of social isolation met Eigenvalue-one criteria and explained 71.0%, 54.5%, 66.0%, 65.0%, 71.0%, 69.4%, 56.0%, and 65.2% of the total variance, respectively. All subscales had a one-factor solution, and all factor loadings were more than twice the critical value of 0.28 [31]. The minimum factor loading was 0.643.

## 4. Discussion

The purpose of this study was to assess the determinants of new technology adoption to promote social connectedness during the COVID-19 pandemic utilizing the conceptual paradigm of MTM. The scientific value of this research lies in its contribution to building evidence-based or theory-based support for developing putative interventions to build social connectedness in the COVID-19 pandemic. As expected, all the three initiation constructs of MTM (participatory dialogue, changes in the physical environment, and behavioral confidence) were statistically significant predictors of the likelihood of initiating new technology use among technology users. These accounted for 38.3% of the variance. Similarly, all the three sustenance constructs of MTM (practice for change, emotional transformation, and changes in the social environment) were statistically significant predictors of the likelihood of continuing new technology use among technology users and accounted for 42.6% of the variance. These findings confirm that the MTM constructs help understand both starting and continuing the use of technology during the COVID-19 pandemic in a nationally representative sample of the population. Our findings reached substantial explanatory power in the behavioral and social sciences [25]. There can be other potential factors that contribute to the performance of any behavior, such as genetics, personality characteristics, irrational beliefs, social norms, policies, etc., that cannot be measured in any given study, thus preventing accountability of predictability to close to 100%. The findings are further supported by modeling conducted with non-technology users in which behavioral confidence and changes in the physical environment were significant contributors along with sex for starting the use of the new technology and accounted for 39.5% of the variance. These were indicative of a positive association in consonance with the theoretical proposition. Similarly, changes in the social environment and sex were significant, accounting for 48.2% of the variance and indicative of a positive association per the theoretical proposition. These findings among non-technology users combined with the findings mentioned above with technology users, lend credibility to MTM as a strong explanatory model on which interventions to promote technology use can be designed. All the constructs of MTM are modifiable, making it easy to translate them into intervention designing and evaluation.

The study also found that social isolation (6.24 ± 3.3) was a problem during the COVID-19 pandemic. While the sample size was limited, a total of 47.1% of the respondents reported having high levels of social isolation (score above 6.0 units on a scale of 0–12). These findings were consistent with reports from Rosenberg and colleagues (2020) that the prevalence of loneliness was 54% during the COVID-19 pandemic in April 2020 [14]. Our study was conducted in March 2021 when restrictions were relatively relaxed, resulting in slightly lower rates of social isolation. While social isolation has been reported as a significant problem during the COVID-19 pandemic [5,12,31], and the use of technology has been suggested as a means to cope with it [15,32,33]. We could not find any systematic studies that linked the use of technology with social isolation or loneliness during the COVID-19 pandemic. Prior to the COVID-19 pandemic, the problem of social isolation and loneliness was still relatively high and was known to have adverse health consequences [34]. Nearly half the population in our sample reported that social isolation was a problem, which underscores the need for rigorous public health efforts. The promotion of new technology can serve as an effective tool in the repertoire of public health professionals. The COVID-19 pandemic has provided an impetus for the promotion of new technology, which should be channeled into future intervention planning.

Regarding the use of new technology, it was found that 40.6% have not used any new technology. This is especially relevant because 93.5% of participants had reported current access to a smartphone with the internet, and 95.5% owned a mobile phone. Smartphones can be used as potent means to promote interventions in the future. Furthermore, this study found a higher mean score of social isolation among technology users than non-technology users. This may be due to a high degree of socially isolated individuals in this group being more motivated to use new technology to connect with others. Our study examined the following types of new technology use: video conferencing, smartphone apps, mHealth, virtual reality, video games, social sharing platforms, and exergames. Since the questionnaire asked the respondents to mark all the options they were using, 40.7% of respondents marked more than one category, followed by the use of video-conferencing alone (12.6%).

A closer examination of each construct of MTM guides health promotion program planning to address social isolation and the role of new technology use. In the initiation model, the construct of behavioral confidence had the largest and statistically significant contribution for technology users and non-technology users, indicating it to be the strongest predictor. This finding is supported by several studies, for example, Yoshany and colleagues (2021) found behavioral confidence to be a significant and strongest predictor in their study of nutritional behaviors among menopausal women [35]. Sharma and colleagues, in their study predicting handwashing behavior, found a significant and strongest behavioral confidence construct in the study sample [21]. Williams and colleagues also found a significant and strongest contribution of behavioral confidence for changes in fruit and vegetable consumption behavior among Black men [36]. Our findings suggest that behavioral confidence must be developed among the general population to use new technology during the COVID-19 and post-pandemic periods to improve social connectedness and reduce social isolation and loneliness. Behavioral confidence can be built in interventions promoting new technology by introducing the learning into small steps, using multiple internal and external sources that infuse confidence, projecting acquisition of behavior change to a future date, and reducing associated stress.

The second construct found to be important in our study for starting the adoption of new technology to improve social connectedness was physical environment changes that entail accessibility and availability of newer technology. This finding is also supported by other studies on MTM with the availability of fruits and vegetables [36] and healthy nutritional options [35]. The construct also aligns with the diffusion of innovations theory construct involving adopting innovations [36,37]. With technology innovations, various environmental factors such as reducing complexity, increasing compatibility, improving demonstrability, reducing costs, and allowing for modifications by the user may be useful aspects to keep in mind for interventions promoting new technology, especially among those experiencing social isolation [23,38].

The construct of participatory dialogue (e.g., the participant is convinced that the positives of using new technology outweigh the negatives of using new technology) was significant for technology users but not for non-technology users. This finding underscores the need for designing interventions that promote the positives aspects of new technology to enhance its adoption among potential users. This finding is also supported by the construct of the relative advantage, or how new technology may appear to be better than other alternatives, as advocated in Roger’s diffusion of innovations theory [37]. Other constructs from this model such as compatibility, reduction of complexity, demonstrability, reduction of costs, and clarity of results may also be important aspects to highlight during participatory dialogue.

For continued use of new technology, the construct of changes in the social environment in MTM was statistically significant for both technology users and non-technology users. The higher values of estimated coefficients indicate the need for continued social support to maintain putative behavior change among non-users. This construct is important in several studies, which tested the applicability of MTM. For example, studies have found that changes in the social environment were important for physical activity behavior change [39], portion size behavior change [22], and fruit and vegetable consumption behavior change [40]. This construct is also important from the perspective of diffusion of innovations theory that emphasizes the construct of the social system. Social networks, change agents, opinion leaders, and person-to-person dissemination are important for adopting innovations such as new technology [37].

The construct of emotional transformation in MTM or directing feelings towards using new technology to connect with others was significant for technology users (β = 0.326, *p* < 0.001) but not for non-technology users. The recognition and regulation of emotions is an essential part of emotional intelligence [41]. This concept is gaining popularity and could be pivotal for promoting the use of technology for social connectedness. In several applications of MTM, this construct has demonstrated significance regarding physical activity behavior change [39], portion size behavior changes [22], and replacing sugar-sweetened beverage consumption with water [42]. The negative emotions of sadness, helplessness, despair, feeling stressed, feeling anxious, and so on can all be channeled into positive applications of applying energy toward learning and using technology to connect with others, especially during the COVID-19 pandemic.

The construct of practice for change in MTM or persistent thinking about using technology to connect with others was significant for technology users (β = 0.243, *p* < 0.001) but not for non-technology users. This construct facilitates the initial adoption of new technology and then supports its continued use [18,23]. In several MTM based studies, this construct is influential in explaining the maintenance of behavior change [23,39,40]. Thus, ample opportunity for practicing and reflecting on the use of technology holds promise for increasing social connectedness and reducing social isolation during the COVID-19 pandemic.

In our study, several demographic characteristics were found to be significant with technology use. For example, there was a significant difference between older and younger populations. We operationalized age as a dichotomous variable comprising those under 55 years of age and those 55 years of age and older. As expected, we found that the use of new technology was significantly lower among those over 55 years of age. Future research on identifying technology use correlations specific to older populations may be necessary for reducing social isolation during the COVID-19 pandemic. There is a need to design different interventions for younger and older populations.

Another demographic characteristic that we found to be significantly different between users and non-users of technology was ethnicity, with fewer Hispanics (21.1%) using new technology (*p* = 0.02). This finding is somewhat contrary to the findings of a study on HIV prevention among Hispanic women that found high levels of comfort with technology use [43], and also a study was performed in New York that a large majority of Hispanics had computers at home and used the internet regularly [44]. Further, as expected, 68.4% of unemployed participants were not using new technology (*p* <0.0001). This could be related to their non-affordability of new technology. Likewise, respondents earning less than $25,000 per year (36.1%) were more non-users. This could also be related to the non-affordability of new technology. There is a need to target some of these subgroups that exhibit greater disparities.

### 4.1. Implications for Practice

There is a need for technology promotion programs at all levels to improve social connectedness to alleviate social isolation during the COVID-19 pandemic. Such programs can be promoted by health education specialists, healthcare providers, health workers, counselors, mental health professionals, public health professionals, policymakers, computer professionals, etc. The new technology can include the utilization of m-Health (i.e., use of mobile phones as part of a health program), smartphone apps (e.g., WhatsApp, Instagram, Facetime, Skype, etc.), virtual reality in groups (e.g., guided meditation in groups using virtual assets), video conferencing (e.g., Zoom, WebEx, etc.), videogames (i.e., multi-player games), exergames using multiple users (i.e., phone or computer-based group exercise in groups), and social sharing platforms (e.g., “My Country Talks”; https://www.mycountrytalks.org) [45].

MTM serves as a useful framework in promoting new technology use. For instance, facilitating behavior change is part of behavioral confidence, which can be built by exploring the sources for enhancing the ability to use technology that appeals to the person. This can come in the form of letting users experiment with newer technology, having YouTube tutorial guides, providing short and simple stepwise guides both online and in technology. Secondly, changes in the physical environment in the form of new technology availability are also important for starting the adoption of new technology. Subsidizing availability, especially for individuals from lower-income backgrounds, should be a priority for policy action.

For the continuation of the use of new technology also MTM constructs can help. The construct of changes in the social environment helps to explain the fostering of social networks, utilizing change agents, mobilizing opinion leaders, and using friends and family members can serve as effective means to promote the continued use of new technology. In previous interventions of MTM, changes in social environment construct have been used to promote behavior change and foster the use of technology [19,46]. The constructs of emotional transformation whereby directing negative feelings into positive ones for the use of technology and practice for change of constant practice of new technology will also go a long way in improving the continued use of technology.

### 4.2. Strengths and Limitations

To our knowledge, this is the first study that looks at theory-based correlates of the use of new technology in the COVID-19 pandemic to improve social connectedness. The study provides evidence that social isolation is becoming a problem in modern times, and new technology can help in this process. The study provides a psychometrically robust instrument that can be used for testing future intervention applications. The utilization of an up-to-date model such as MTM can help in adopting new technology. There are also several limitations of the study. A cross-sectional study limits the establishment of causal inferences due to data on independent correlations and dependent variables being collected simultaneously. Further, reliance on self-reports introduces potential measurement bias. Next, there can be other potential factors, including genetics, personality characteristics, irrational beliefs, social norms, policies, which may affect the performance of the behavior change and cannot be measured in any given study. These unmeasured variables may prevent accountability of predictability to close to 100%. Finally, even though we collected data from a nationally representative sample in terms of gender and race, all other variables, including age and region/geographical distribution, could have introduced sampling bias, which limits the generalizability of our findings. Moreover, the sole purpose of this study was model testing and did not determine the prevalence estimates. Moreover, COVID-19 restrictions posed challenges in the sampling. Future studies with a relatively bigger sample size can be planned to estimate prevalence.

## 5. Conclusions

During the COVID-19 pandemic, social isolation has grown, and there is a need to improve social connectedness through new technology. The study provides evidence that MTM is a useful model in explaining the promotion and adoption of new technology to address the issue of social isolation and promoting social connectedness. Future research should discern the determinants of social connectedness based on MTM for various subgroups based on factors such as age, race/ethnicity, employment status, etc. There is an ardent need to design and test the efficacy of interventions based on MTM that can be utilized to promote social connectedness through the use of new technology. In summary, MTM can lead the way for evidence-based intervention planning in this regard.

## Figures and Tables

**Table 1:** Descriptive statistics of the study population (*n* = 382).

Variable	Characteristics	Mean ± SD	n (%)
Age	-	43.9 ± 18.3	-
Sex	Female	-	192 (50.3)
	Male	-	189 (49.5)
Race	White/Caucasian	-	272 (71.2)
	Non-white	-	110 (28.8)
Ethnicity	Hispanic	-	66 (17.3)
	Non-Hispanic	-	316 (82.7)
Employment	Yes	-	177 (46.3)
	No	-	205 (53.7)
Number of hours worked weekly * Income	-	36.1 ± 25.3	-
	<$25,000	-	99 (25.9)
	$25,001–$50,000	-	92 (24.1)
	$50,001–$75,000	-	77 (20.2)
	$75,001–$100,000	-	37 (9.7)
	$100,001–$125,000	-	24 (6.3)
	>$125,000	-	33 (8.7)
	Prefer not to answer	-	20 (5.2)
Residence	Rural	-	111(29.1)
	Semiurban	-	129 (33.8)
	Urban	-	142 (37.1)
Health insurance	Yes	-	325 (85.1)
	No	-	57 (14.9)
Marital status	Married	-	170 (44.5)
	Never married	-	117 (30.6)
	Divorced/separated	-	43 (11.3)
	Widowed	-	16 (4.2)
	Others **	-	36 (9.4)
Smartphone with internet	Yes	-	357 (93.5)
	No	-	25 (6.5)
Used new technology during COVID-19	Yes	-	227 (59.4)
	No	-	155 (40.6)
Social isolation	Low (score ≤6.0)	-	202 (52.9)
	High (score > 6.0)	-	180 (47.1)
Type of technology used	Video conferencing	-	48 (12.6)
	Smartphone apps	-	36 (9.4)
	M-health	-	11 (2.9)
	Other ***	-	33 (8.7)
	More than one (the combination of the above)	-	155 (40.6)
	None	-	99 (25.9)
Mobile phone	Yes	-	365 (95.5)
	No	-	17 (4.5)

* Number of hours were reported by 169 (44.2%) participants only. ** Other categories include a member of unmarried couple+ registered domestic partnership. *** Other categories in a type of technology include Virtual reality, video games, social sharing platforms, and exergames.

**Table 2:** Comparison of categories across technology users and non-users, (*n* = 382).

Variable	Characteristics	New Technology Use During COVID-19 *n* (%)		*p*-Value
		(Yes, *n* = 227, 59.4%)	(No, *n* = 155, 40.6%)	
Age groups	<55 years	166 (73.1)	95 (61.3)	0.02
	≥55 years	61 (26.9)	60 (38.7)	-
Sex	Female	119 (52.4)	73 (47.1)	0.4
	Male	107 (47.1)	82 (52.9)	-
Race	White/Caucasian	162 (71.4)	110 (71.0)	0.9
	Non-white	65 (28.6)	45 (29.0)	-
Ethnicity	Hispanic	48 (21.1)	18 (11.6)	0.02
	Non-Hispanic	179 (78.9)	137 (88.4)	-
Employment	Yes	128 (56.4)	49 (31.6)	<0.0001
	No	99 (43.6)	106 (68.4)	-
Income	<$25,000	43 (18.9)	56 (36.1)	<0.0001 *
	$25,001–$50,000	52 (22.9)	40 (25.8)	0.5
	$50,001–$75,000	51 (22.5)	26 (16.8)	0.2
	$75,001–$100,000	23 (10.1)	14 (9.0)	0.7
	$100,001–$125,000	20 (8.8)	4 (2.6)	0.6
	>$125,000	28 (12.3)	5 (3.2)	<0.0001 *
Residence	Rural	58 (25.6)	53 (34.2)	0.1
	Semiurban	77 (33.9)	52 (33.5)	-
	Urban	92 (40.5)	50 (32.3)	-
Health insurance	Yes	201 (88.5)	124 (80.0)	0.02
	No	26 (11.5)	31 (20.0)	-
Marital status	Married	106 (46.7)	64 (41.3)	0.5
	Never married	69 (30.4)	48 (31.0)	-
	Divorced/Separated	21 (9.3)	22 (14.2)	-
	Widowed	8 (3.5)	8 (5.2)	-
	Others	23 (10.1)	13 (8.4)	-
Social isolation	Low (score ≤ 6.0)	103 (45.4)	99 (63.9)	<0.0001
	High (score > 6.0)	124 (54.6)	56 (36.1)	-
Smartphone with internet	Yes	219 (96.5)	138 (89.0)	0.004
	No	8 (3.5)	17 (11.0)	-
Mobile phone	Yes	223 (98.2)	142 (91.6)	0.002
	No	4 (1.8)	13 (8.4)	-

* *p*-values in multiple comparisons are Bonferroni corrected.

**Table 3:** Comparing mean scores of MTM constructs and reliability diagnostics across groups.

Groups	Those Who Used Technology During COVID-19 (*n* = 227)				Those Who Did Not Use Technology During COVID-19 (*n* = 155)				
Constructs	Possible Score Range	Observed Score Range	Mean ± SD	Cronbach’s Alpha	Possible Score Range	Observed Score Range	Mean ± SD	Cronbach’s Alpha	*p*-Value *
Initiation	0–4	0–4	2.72± 1.2	-	0–4	0–4	1.82 ± 1.3	-	<0.0001
Social isolation	0–12	0–12	6.96 ± 3.0	0.83	0–12	0–12	5.51 ± 3.6	0.83	<0.0001
Participatory dialogue: advantages	0–12	0–12	7.16 ± 2.79	0.83	0–12	0–12	4.77 ± 3.3	0.90	<0.0001
Participatory dialogue: disadvantages	0–12	0–12	4.68 ± 3.11	0.79	0–12	0–12	4.68 ± 3.13	0.77	0.9
Participatory dialogue **	−12–+12	−8–+12	2.48 ± 3.4	-	−12–+12	−12–+10	0.09 ± 3.8	-	<0.0001
Behavior confidence	0–12	0–12	8.25± 2.87	0.81	0–12	0–12	6.48 ± 3.4	0.87	<0.0001
Changes in the physical environment	0–12	0–12	7.61 ± 3.1	0.81	0–12	0–12	5.72 ± 3.5	0.86	<0.0001
Entire initiation scale	-	-	-	0.82	-	-	-	0.84	-
Sustenance	0–4	0–4	2.78 ± 1.09	-	0–4	0–4	1.99 ± 1.23	-	<0.0001
Emotional transformation	0–12	0–12	7.48± 3.05	0.85	0–12	0–12	5.63 ± 3.45	0.89	<0.0001
Practice for change	0–12	0–12	7.43 ± 3.04	0.83	0–12	0–12	5.80 ± 3.58	0.90	<0.0001
Changes in the social environment	0–12	0–12	7.49 ± 2.98	0.73	0–12	0–12	5.75 ± 3.46	0.81	<0.0001
Entire sustenance scale	-	-	-	0.90	-	-	-	0.94	-
Entire scale	-	-	-	0.91	-	-	-	0.93	-

* *p*-values of independent-samples-t test ** participatory dialogue (advantages-disadvantages).

**Table 4:** Predicting likelihood for initiation and sustenance of technology users (*n* = 227) through HRM.

Variables	Model 1		Model 2		Model 3		Model 4	
	B	β	B	β	B	β	B	β
**The Likelihood for Initiation as a Dependent Variable**								
Constant	2.34 **	-	2.23 **	-	1.22 **	-	0.59	-
Age	−0.074	−0.028	−0.176	−0.067	−0.015	−0.006	−0.016	−0.006
Sex	−0.130	−0.056	−0.107	−0.046	−0.155	−0.067	−0.140	−0.060
Income	0.054	0.083	0.059	0.090	−0.012	−0.019	0.003	0.005
Social isolation	0.056	0.146	0.046	0.119	0.023	0.059	0.017	0.043
Participatory dialogue	-	-	0.105 **	0.307	0.056 **	0.163	0.054 *	0.158
Changes in the physical environment	-	-	-	-	0.176 **	0.472	0.053 *	0.143
Behavioral confidence	-	-	-	-	-	-	0.184 **	0.455
R^2^	0.038	-	0.130	-	0.310	-	0.408	-
F	1.43	-	4.64 **	-	12.19 **	-	16.55 **	-
ΔR^2^	0.038	-	0.092	-	0.180	-	0.098	-
ΔF^2^	1.43	-	23.03 **	-	56.75 **	-	35.74 **	-
**The Likelihood for Sustenance as a Dependent Variable**								
Constant	2.51 **	-	1.12 **	-	0.94 **	-	0.80 **	-
Age	−0.011	−0.005	0.059	0.024	0.122	0.050	0.167	0.069
Sex	−0.161	−0.075	−0.153	−0.071	−0.165	−0.076	−0.161	−0.074
Income	0.035	0.059	0.005	0.008	−0.003	−0.005	−0.017	−0.027
Social isolation	0.029	0.082	−0.005	−0.014	−0.005	−0.014	−0.012	−0.032
Emotional transformation	-	-	0.218 **	0.619	0.138 **	0.390	0.115 **	0.326
Practice for change	-	-	-	-	0.100 **	0.282	0.086 **	0.243
Changes in the social environment	-	-	-	-	-	-	0.061 *	0.169
R^2^	0.040	-	0.408	-	0.433	-	0.449	-
F	1.507	-	21.46 **	-	20.68 **	-	19.55 **	-
ΔR^2^	0.040	-	0.368	-	0.025	-	0.016	-
ΔF^2^	1.507	-	135.64 **	-	9.40 **	-	6.389 *	-

B (Unstandardized coefficient); β (Standardized coefficient), * *p*-value < 0.05; ** *p*-value < 0.001.

**Table 5:** Predicting likelihood for initiation and sustenance of technology non-users (*n* = 155) through HRM.

Variables	Model 1		Model 2		Model 3		Model 4	
	B	β	B	β	B	β	B	β
**The Likelihood For Initiation As A Dependent Variable**								
Constant	1.965 **	-	1.899 **	-	0.909 *	-	0.596	-
Age	−0.466 *	−0.179	−0.450*	−0.172	−0.317	−0.121	−0.286	−0.110
Sex	0.459 *	0.180	0.567 **	0.222	0.358 *	0.140	0.372 *	0.146
Income	0.066	0.090	0.063	0.085	0.025	0.035	0.036	0.049
Social isolation	0.033	0.095	0.021	0.059	−0.009	−0.027	−0.005	−0.014
Participatory dialogue	-	-	0.099 **	0.030	0.051 *	0.154	0.039	0.117
Changes in the physical environment	-	-	-	-	0.190 **	0.526	0.109 **	0.300
Behavioral confidence	-	-	-	-	-	-	0.109 **	0.293
R^2^	0.083	-	0.166	-	0.400	-	0.430	-
F	2.23 *	-	4.18 **	-	12.17 **	-	12.18 **	-
ΔR^2^	0.083	-	0.083	-	0.234	-	0.030	-
ΔF^2^	2.23 *	-	14.61 **	-	56.99 **	-	7.76 **	-
**The likelihood for Sustenance as a dependent variable**								
Constant	1.639 **	-	0.092	-	0.052	-	0.006	-
Age	−0.221	−0.087	0.103	0.041	0.079	0.031	0.030	0.012
Sex	0.443 *	0.180	0.288	0.117	0.311	0.126	0.323 *	0.131
Income	0.018	0.025	−0.023	−0.033	−0.026	−0.037	−0.041	−0.058
Social isolation	0.072 **	0.211	0.057 *	0.167	0.54 *	0.159 *	0.048	0.141
Emotional transformation	-	-	0.215 **	0.602	0.106 *	0.295	0.052	0.144
Practice for change	-	-	-	-	0.117 *	0.341	0.072	0.208
Changes in the social environment	-	-	-	-	-	-	0.140 **	0.393
R^2^	0.092	-	0.417	-	0.439	-	0.513	-
F	2.495 *	-	15.05 **	-	14.3 **	-	16.94 **	-
ΔR^2^	0.092	-	0.326	-	0.021	-	0.074	-
ΔF^2^	2.50 *	-	82.5 **	-	5.55 *	-	21.94 **	-

* *p*-value < 0.05; ** *p*-value < 0.001.

## Data Availability

The data presented in this study are available on request from the corresponding author. The data are not publicly available due to ethical reasons.
